# Primary cell culture and characterization of a pleomorphic adenoma. A case report

**DOI:** 10.21142/2523-2754-1301-2025-234

**Published:** 2025-03-03

**Authors:** Selenne Romero-Servin, Nancy Leticia Mendoza-Martínez, Francisco Germán Villanueva-Sánchez, Ilse Patricia Rodríguez-Tapia, Aylin Divina Cadena-Galeana, René García-Contreras

**Affiliations:** 1 Interdisciplinary Research Laboratory (LII), Oral and Maxillofacial Pathology area, National School of Higher Studies (ENES) Leon, National Autonomous University of Mexico. Leon, Mexico. selenneservin@gmail.com nancy.camille18@gmail.com fgvillanuevas@enes.unam.mx ilsertapia@gmail.com aylincadenag@gmail.com Universidad Nacional Autónoma de México Interdisciplinary Research Laboratory (LII) Oral and Maxillofacial Pathology area, National School of Higher Studies (ENES) Leon National Autonomous University of Mexico Leon Mexico selenneservin@gmail.com nancy.camille18@gmail.com fgvillanuevas@enes.unam.mx ilsertapia@gmail.com aylincadenag@gmail.com; 2 Interdisciplinary Research Laboratory (LII); Nanostructures and Biomaterials area, National School of Higher Studies (ENES) Leon, National Autonomous University of Mexico. leon, Mexico. rgarciac@enes.unam.mx Universidad Nacional Autónoma de México Interdisciplinary Research Laboratory (LII) Nanostructures and Biomaterials area, National School of Higher Studies (ENES) Leon National Autonomous University of Mexico leon Mexico rgarciac@enes.unam.mx

**Keywords:** pleomorphic adenoma, salivary glands, primary cell culture, salivary gland tumors, immunomarkers, adenoma pleomórfico, glándulas salivales, cultivo celular primario, tumores de glándulas salivales, inmunomarcadores

## Abstract

**Objective:**

This case report aimed to characterize primary pleomorphic adenoma cells obtained from a parotid gland tumor through tissue biopsy, primary cell culture, and immunohistochemical analysis.

**Methods:**

A tissue biopsy sample from a 58-year-old patient with pleomorphic adenoma underwent histopathological examination and primary cell culture. The primary cells were characterized through immunohistochemical staining using antibodies against S-100, SMA, Vimentin, and cytokeratin AE1/AE3. Additionally, paraffin-embedded tissue sections were stained using cytokeratin AE1/AE3, Vimentin, Calponin, SMA, S100, and p63.

**Results:**

Primary cell culture revealed weak S-100 staining and positive SMA in myoepithelial cells, while Vimentin and AE1/AE3 were negative in all the cell population. In the paraffin-embedded tissue, cytokeratin exhibited strong cytoplasmic and membranous positivity in luminal cells. Vimentin showed cytoplasmic staining in myoepithelial cells. S-100 displayed weak nuclear and strong cytoplasmic immunoreactivity in myoepithelial cells. SMA presented weak membranous positivity in a few myoepithelial cells, and finally, p63 showed nuclear staining in abluminal cells. Calponin showed negative staining in neoplastic cells and stroma.

**Conclusion:**

The results showed that primary component of pleomorphic adenoma comprises myoepithelial cells. The identification of cell cultured *in vitro* is pivotal in comprehending the cellular components of these neoplasms. This comprehensive characterization of primary pleomorphic adenoma cells provides insights into their morphology, immunophenotype, and histological features.

## RESUMEN

**Objetivo:**

Este reporte de caso tuvo como objetivo caracterizar las células primarias del adenoma pleomórfico obtenidas de un tumor de glándula parótida mediante biopsia de tejido, cultivo celular primario y análisis inmunohistoquímico.

**Métodos:**

Una muestra de biopsia de tejido de un paciente de 58 años con adenoma pleomórfico se sometió a examen histopatológico y cultivo celular primario. Las células primarias se caracterizaron mediante tinción inmunohistoquímica utilizando anticuerpos contra S-100, SMA, vimentina y citoqueratina AE1/AE3. Además, se tiñeron secciones de tejido embebido en parafina utilizando citoqueratina AE1/AE3, vimentina, calponina, SMA, S100 y p63.

**Resultados:**

El cultivo celular primario reveló tinción débil de S-100 y SMA positivo en células mioepiteliales, mientras que vimentina y AE1/AE3 fueron negativas en toda la población celular. En el tejido embebido en parafina, la citoqueratina exhibió positividad citoplasmática y membranosa fuerte en células luminales. La vimentina mostró tinción citoplasmática en células mioepiteliales. S-100 mostró inmunorreactividad nuclear débil y citoplasmática fuerte en células mioepiteliales. SMA presentó positividad membranosa débil en algunas células mioepiteliales y, finalmente, p63 mostró tinción nuclear en células abluminales. La calponina mostró tinción negativa en células neoplásicas y estroma.

**Conclusión:**

Los resultados mostraron que el componente primario del adenoma pleomórfico comprende células mioepiteliales. La identificación de células cultivadas *in vitro* es fundamental para comprender los componentes celulares de estos tumores. Esta caracterización integral de las células primarias del adenoma pleomórfico proporciona información sobre su morfología, inmunofenotipo y características histológicas.

## INTRODUCTION

Salivary glands are classified into two primary groups: the minor and major salivary glands (SGs) ^1^. In humans, the major salivary glands comprise three pairs: the parotid glands (PG), submandibular glands (SMG), and sublingual glands (SLG). Additionally, there has been recent discussion regarding the identification of a fourth major salivary gland, or a distinct organ known as the tubarial gland, which represents macroscopic glandular tissue sites of clinical significance ^2^^,^^3^. All salivary glands share a fundamental anatomical structure characterized by branched ducts that release into the oral cavity, accompanied by secretory units called acini, which produce saliva. Both major and minor salivary glands are composed of acinar cells, myoepithelial cells, and ductal cells, supported by blood vessels and nerves network ^4^. The loss of homeostasis in the function of the salivary glands leads to neoplasia in these structures, with pleomorphic adenoma being the most frequent.

Neoplasms affecting the salivary glands present a unique category within head and neck tumors, characterized by their infrequent appearance, diverse histologic subtypes, and often overlapping characteristics ^5^. These tumors, ranging from 7.03 to 8.58 per 100,000 individuals, with an annual occurrence rate, constitute rare malignancies in the head and neck region ^6^.

Pleomorphic adenoma (PA) represents the most frequently encountered tumor affecting the salivary glands. This tumor is characterized by a mixture of epithelial and mesenchymal components ^7^. Also referred to as a mixed tumor, PA accounts for the majority, approximately 81.2%, of benign neoplasms originating from the salivary glands ^8^. It predominantly occurs in individuals aged between 30 and 60 years, with a noted female predominance according to literature reports ^9^.

From a histological perspective, PA typically exhibits a diverse epithelial pattern within a matrix composed of loose fibrous, myxoid, chondroid, or mucoid stroma. This histologic recognition of PA is characterized by primary components: epithelial cells, myoepithelial cells, and mesenchymal elements ^10^.

Pleomorphic adenoma is known for its high morphological plasticity, primarily attributed to the myoepithelial component ^11^. While PA remains the most common salivary gland neoplasm, there have been limited instances of cell cultures conducted to facilitate their subculture and characterization. Cell cultures have been employed to identify the myoepithelial component, assess its function, and explore its role in epithelial-mesenchymal transition. 

Various immunomarkers have been utilized to identify components of PA including cytokeratins for luminal epithelial cells, p40/p53 for basal and outer abluminal myoepithelial cells, and specific myoepithelial cell markers such as SMA, Calponin, S100 and SOX10. These tests have predominantly been conducted using paraffin-embedded tissue blocks, while reports on *in vitro* characterization are sparse in the literature. 

Few *ex vivo* studies have been undertaken to characterize the cellular diversity of PA. Hence, the identification of primary cell cultures *in vitro* was crucial for understanding the cellular components and immunophenotype of these neoplasms. This justifies the isolation of explants for primary cell culture techniques and subsequent immunohistochemical characterization. This approach allows us to assess and attempt to comprehend cellular behavior and make comparisons with histological tissue.

Therefore, this study sought to comprehensively characterize primary pleomorphic adenoma cells extracted from a parotid gland tumor via tissue biopsy, primary cell culture, and immunohistochemical analysis.

## METHODS

### Tissue biopsy sample

The specimen was collected from a 58-year-old individual with a parotid gland tumor. The protocol for cellular isolation was authorized by the bioethical committee at ENES Leon Unit, UNAM, under registration code CE_16 004_SN. This authorization was obtained in accordance with the guidelines of the Intramural Board of Ethics Committee. Prior to tissue collection, informed consent from the patient was obtained, which included statements acknowledging the use of tissues for research purposes, confidentiality of identity, potential risks, storage of samples, and the right to withdraw consent. The patient had previously been diagnosed with pleomorphic adenoma, though an incisional, and the tumor was surgically removed at the Department of Maxillofacial Surgery form the General Hospital, Leon, Mexico. The soft tissue specimen measured 7.3x12.69x1.69 cm. It was divided into two sections for different analyses: one half underwent histopathological examination, while the other half was utilized for primary cell culture. Both analyses were conducted at the Interdisciplinary Research Laboratory, ENES Leon Unit, UNAM.

### Primary cell culture

Fifty percent of the obtained tissue was stored in a Falcon tube with a solution containing phosphate buffer saline solution (PBS) and 1% of antibiotics (Pen-Strep, 10,000 IU penicillin/10,000 µg/ml of streptomycin, Sigma-Aldrich, St. Lois, MO, USA) for tissue transportation. The procedure was conducted within a horizontal clean laminar flow bench (Biobase, Shandong, China). The tissue was washed three times with PBS, then the explants, measuring 0.5x0.5 mm, were obtained from the sample and placed in tubes with 1ml of 0.05% trypsin in EDTA-2Na (Sigma-Aldrich, St. Lois, MO, USA) and incubated at 37º C, 5% CO2 and 95% of humidity. These tubes were then incubated for 60 minutes, during which the samples were checked and shaken every 20 minutes. After incubation, the samples underwent centrifugation at 5,000 rpm for 10 minutes. After removing the resulting supernatant, the pellet containing the cultured cells was retained. The explants were resuspended in Minimum Essential Medium Eagle (MEM, Sigma-Aldrich, St. Louis, MO, USA) enriched with 20% fetal bovine serum (FBS, Sigma-Aldrich, St. Louis, MO, USA), 1% glutamine (Sigma-Aldrich, St. Louis, MO, USA), and 1% antibiotics. Subsequently, the explants were inoculated into 10-cm cell culture dishes and incubated at 37°C with 5% CO2 for 21 days until reaching 80% confluence. 

### Cell characterization

The primary pleomorphic adenoma cells showed a fusiform like shape, focal adhesion, and cell proliferation. Then, the cells were detached using 0.05% trypsin suspended EDTA-2Na in PBS. Cells at their four population doubling levels (PDL) were placed electrocharged cover slide (BSB7028, Bio SB Hydrophilic Plus Slides, California, USA) at 1x106 cells/ml until reaching 100% confluence to perform the immunohistochemical analysis using S-100, SMA, Vimentin, cytokeratin AE1/AE3 antibodies.

### Immunohistochemical staining of the culture

The staining was conducted on slides. The samples were air-dried for 2 hours at 58°C and then subjected to a dehydration and rehydration process. Slides were placed in a coplin staining jar preheated with ImmunoDNA Retriever, which included citrate or EDTA, in a steamer, and covered in steam for 30 to 60 minutes. Following heat treatment, slides were placed in an ImmunoDNA Retriever containing either citrate or EDTA buffer and allowed to settle for 15 to 20 minutes at the specified temperature. The antibodies were subsequently applied and left at room temperature.

The slides were washed with deionized water, following which the immunohistochemical protocol was continued as per the manufacturer’s instructions. The slides were subsequently incubated with the primary antibodies, mouse monoclonal antibody S-100 (Clone 4C4.9, BioSB, USA,1:200), monoclonal mouse Anti-human Smooth Muscle Actin SMA (Clone 1A4, Dako, USA, 1:75), rabbit monoclonal antibody P63 (Clone EP174, BioSB, USA, 1:100), rabbit monoclonal antibody Vimentin (Clone EP21, Bio SB, USA, 1:100), mouse monoclonal antibodies Cytokeratin cocktail AE1 & AE3 (Clone AE1 & AE3, BioSB, USA, 1:250), mouse monoclonal antibodies Calponin (Clone 26A11, Leica, USA, ready to use). Afterward, the sections were counterstained with hematoxylin, dehydrated, and then mounted.

### Histopathological findings

A 50% tissue biopsy was initially immersed in buffered formalin (4%) for 24 hours. Subsequently, it underwent processing using a histology tissue processor (Histokinnete, Leica, Wetzlar, Germany), followed by embedding in paraffin. After refrigeration at 4°C for 30 minutes, the sample was sectioned into 3-micron slices using a microtome (Leica, Wetzlar, Germany). These sections were then affixed to glass slides, immersed in a water bath, and placed on a hot plate for 1 hour. Subsequently, a staining protocol was applied as follows: immersion in xylene (100%) for 20 minutes, followed by immersion in alcohol (100%, 96%) for 30 seconds. The sections were then stained with hematoxylin for 10 minutes, treated with lithium carbonate for 15 seconds, and subsequently stained with eosin for 10 minutes. After brief immersion in alcohol (96%, 100%) for 10 seconds, the sections were dehydrated in xylene (100%) for 20 minutes. Finally, samples were fixed using resin (El Crisol, Mexico) and coverslipped for examination under light microscopy at 40× magnification (Leica, Wetzlar, Germany). These sections were treated with hematoxylin and eosin (H&E) for light microscope examination.

### Immunohistochemistry staining paraffin-block

The block containing paraffin-embedded tissue was submitted to immunohistochemistry for cytokeratin AE1/AE3, S-100, Vimentin, p63, SMA, calponin. Sections 3 um were made. The samples were treated and incubated with primary antibodies: mouse monoclonal antibody S-100 (Clone 4C4.9, BioSB, USA,1:200), monoclonal mouse Anti-Human Smooth Muscle Actin SMA (Clone 1A4, Dako, USA, 1:75), rabbit monoclonal antibody P63 (Clone EP174, BioSB, USA, 1:100), rabbit monoclonal antibody Vimentin (Clone EP21, Bio SB, USA, 1:100), mouse monoclonal antibodies Cytokeratin cocktail AE1 & AE3 (Clone AE1 & AE3, BioSB, USA, 1:250), mouse monoclonal antibodies Calponin (Clone 26A11, Leica, USA, ready to use).

### Histological evaluation and processing

Two oral and maxillofacial pathologists, experienced in salivary glands, reviewed the slides using the H&E technique. They evaluated the histopathological components of the tumor tissue and provided the final diagnosis of pleomorphic adenoma. The processed slides were scanned using the MoticEasyScan system (MoticEasyScan one, Motic, Hong Kong, China). During the scanning process, the slides were observed through the Pathology image management software (P-IMS, Pathomation BV, Berchem, Belgium). For image processing and cell counting, the Fiji/Image J (Wayne Rasband, NIH, USA) program was employed. Each slide was verified to contain a corresponding positive control sample.

## RESULTS

### Primary cell culture and immunohistochemical staining

**Figure 1:**
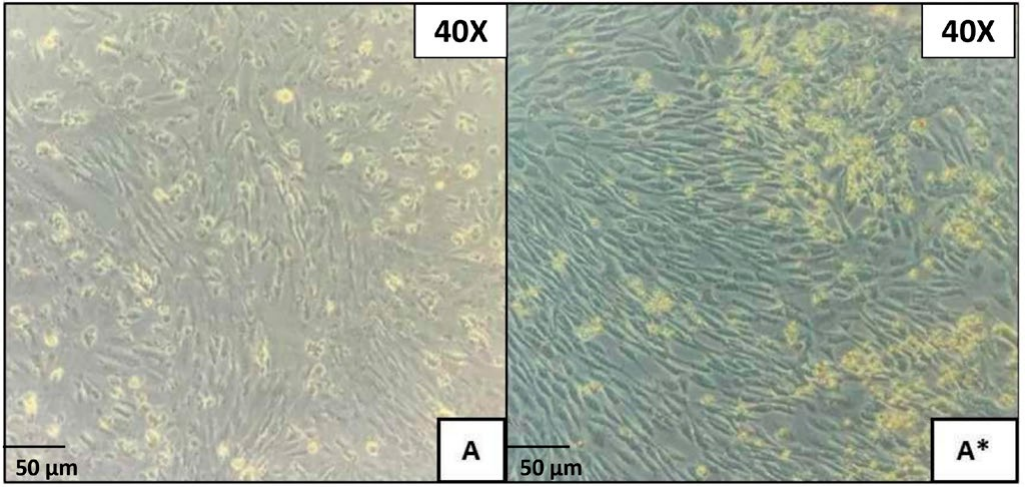
Primary cell culture. Microphotograph A and A*) corresponding of primary cell culture. Incubation was carried out at 37ºC with 5% CO2 for 14 days, until a confluence of 80% was obtained.

**Figure 2:**
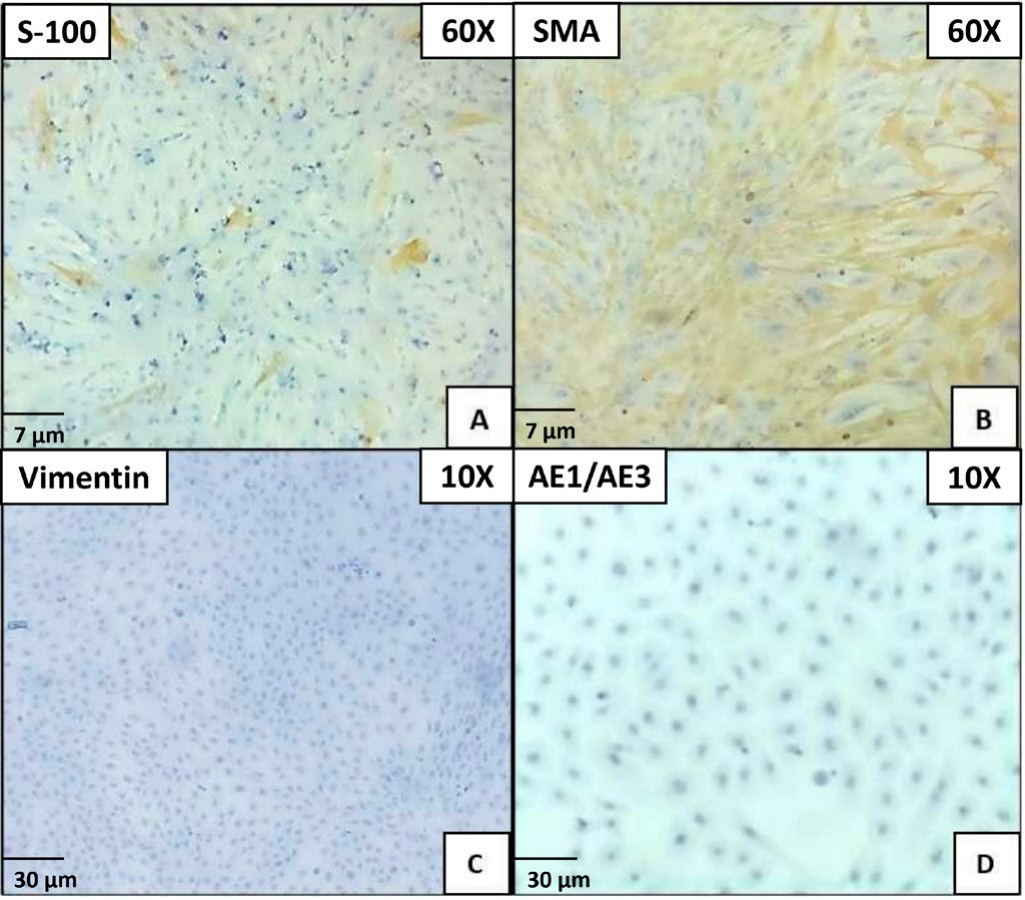
Primary cell culture characterization. Microphotograph A) S-100, viewed at 40x. Star-shaped cells with weak staining. Microphotograph B) SMA viewed at 40x. Cells with spindle-shaped and star-shaped morphology. Cytoplasmatic staining. Microphotograph C) Vimentin viewed at 10x. Negative staining in polygonal cells and microphotograph D) AE1/AE3 viewed at 10x. Negative staining cells with polygonal morphology.

The cells were subcultured successfully, displaying a fusiform morphology, adhering firmly to the polystyrene culture plate, with cellular contact resulting in unidirectional alignment, achieving over 80% cell confluence (Figure 1). The immunohistochemical staining with S-100 exhibited weak staining in some fusiform cells, while the immunomarker SMA showed cytoplasmic positivity in myoepithelial cells with fusiform and stellate morphologies. Vimentin and AE1/AE3 were negative in all cellular populations (Figure 2).

### Immunohistochemistry staining pleomorphic adenoma on paraffin-block

Histopathologically, pleomorphic adenoma exhibits two primary cellular arrangements: one characterized by glandular structures constituted of internal duct epithelium-like cells surrounded by external myoepithelium-like cells, and the other featuring stellate/spindle-shaped cells dispersed within a background of myxochondroid stroma.

**Figure 3:**
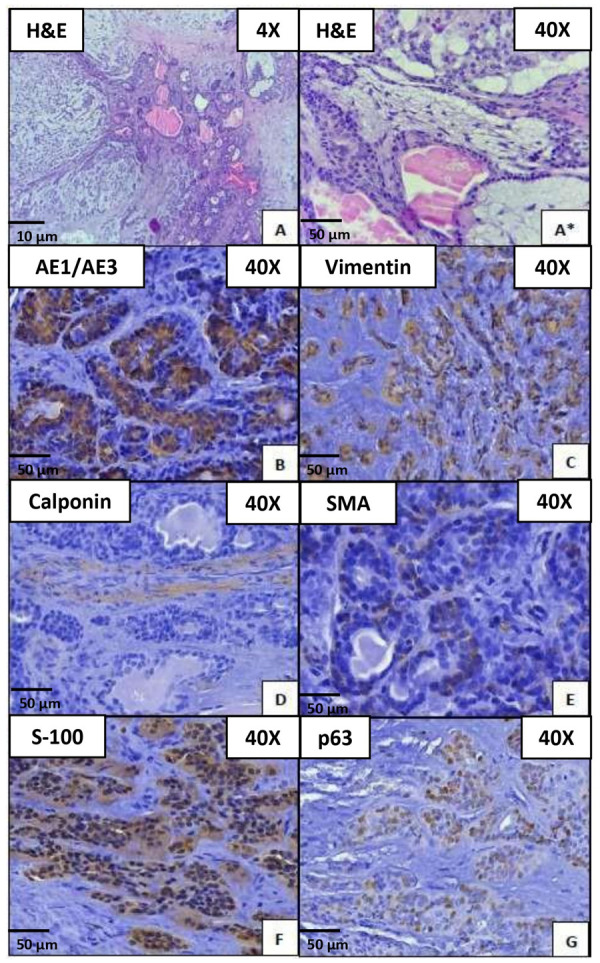
Pleomorphic adenoma on Paraffin-Embedded Blocks. Microphotograph A) H&E viewed at 4x. Neoplasm of salivary gland origin composed of myxoid and chondroid stroma. Ducts containing eosinophilic material within. Microphotograph A*). H&E viewed at 40x. Discohesive epithelial cells forming ducts in a myxoid stroma. Microphotograph B) AE1/AE3. Cytoplasmic and membranous positivity in neoplastic epithelial cells. Microphotograph C) Vimentin viewed at 40x. Cytoplasmatic and focal positivity in cordons and nest with polygonal cells shape. Microphotograph D) Calponin viewed at 40x is negative staining. Microphotograph E) SMA viewed at 40x. Weak membranous positivity in myoepithelial cells. Microphotograph F) S-100 viewed at 40X. Nuclear and cytoplasmatic focal immunoreactivity in neoplastic parenchymal cells. Microphotograph G) p63 viewed at 40x. Nuclear staining for non-luminal tumor cells.

The immunomarker cytokeratin AE1/AE3 exhibited strong cytoplasmic and membranous positivity in luminal cells. Vimentin showed cytoplasmic staining in nest and short cordons of myoepithelial cells. S-100 displayed weak nuclear and strong cytoplasmic immunoreactivity in myoepithelial cells with a polygonal shape, while Calponin showed negative staining in neoplastic cells and stroma. SMA presented weak membranous positivity in a few myoepithelial cells, and finally, p63 showed nuclear staining in abluminal cells (Figure 3).

## DISCUSSION

Salivary gland neoplasms represent a distinct subset among head and neck tumors, characterized by their relatively infrequent incidence, wide spectrum of histologic subtypes, and frequent overlap in features. The histological composition of salivary glands can be divided into two main sections: luminal cells, which encompass acinar and ductal cells, and abluminal cells, consisting of myoepithelial and basal cells ^5^.

Pleomorphic adenomas (PMAs) represent the predominant type, accounting for approximately 86% of benign tumors originating in the salivary glands, thus standing as the most prevalent within this category ^6^. Also known as mixed tumors ^8^, pleomorphic adenomas (PA) are characterized by a mixture of epithelial and mesenchymal components ^7^. The term 'pleomorphic adenoma' reflects the structural complexity of the tumor, which varies among individuals and glands. PA presents distinctive histopathologic characters, originating from a single cell that discerns into epithelial or myoepithelial cells, rather than concurrent proliferation of carcinogenic cells from both epithelium and myoepithelium ^9^.

PA is distinguished by a significant morphological plasticity, primarily attributed to the myoepithelial component ^11^. It might appear that, during neoplastic transformation, myoepithelial cells undergo changes in immunophenotype, losing reactivity for certain markers and gaining expression of others ^11^. Moreover, these lesions frequently pose a diagnostic challenge for pathologists because of the overlapping morphological features they exhibit ^1^.

Studies have indicated variable immunoreactivity among myoepithelial cells in salivary gland pleomorphic adenomas, making it challenging to identify a specific strict marker for them ^11^. The diagnosis relying on hematoxylin and eosin staining techniques continues to be regarded as the gold standard in salivary gland pathology ^6^.

PA typically exhibits a diverse epithelial pattern within a matrix composed of loose fibrous, myxoid, chondroid, or mucoid-type stroma. The identification of PA involves recognizing three primary components: epithelial, myoepithelial, and mesenchymal ^10^.

Myoepithelial cells (MECs) serve various functions including epithelial cell differentiation, contractile function, sensory roles, preservation of gland patency, metabolite transference, formation, and upkeep of the basement membrane, as well as tumor suppression ^12^. The established role of MECs in salivary gland tumors is widely acknowledged. It is recognized that MECs have the capability to differentiate into both epithelial and mesenchymal components within a tumor, and these cells are termed neoplastic myoepithelial cells (NMECs). The capacity of NMECs to specify a mesenchymal component to salivary gland tumors may be attributed to their resemblance to smooth muscle features exhibited by MECs ^13^. These cells can appear in diverse cytological forms, including hyaline, myxoid, or epithelial cells. However, they commonly exhibit angular, subtly separated appearances surrounding ducts or forming clusters of differing sizes or sheet-like structures. Importantly, they do not display the typical characteristics of normal myoepithelial cells. The range of myoepithelial cell varieties produced by neoplastic myoepithelial cells (NMECs) includes myxoid, chondroid, myxochondroid, fibrous, elastic, and even osteoid types ^13^.

About 70% of salivary gland tumors demonstrate myoepithelial cell differentiation and are subsequently categorized with the presence or absence of luminal cell diversity. Tumors lacking luminal cell differentiation are classified as either myoepithelioma or myoepithelial carcinoma ^14^. Approximately 70% of salivary gland tumors demonstrate differentiation into myoepithelial cells, and their classification depends on the presence or absence of luminal cell differentiation. Tumors lacking luminal cell differentiation are further designated as either myoepithelioma or myoepithelial carcinoma. Neoplastic ductal cells organize into glands, with their luminal surface (apical portion) showing positivity for epithelial membrane antigen (EMA) and carcinoembryonic antigen (CEA). Calponin, alongside α-smooth muscle actin (α-SMA), acts as a highly specific marker, although sporadic weak and non-specific signals for calponin may be evident in ductal cells. While S-100 protein and vimentin are highly sensitive markers for neoplastic myoepithelial cells, they are also commonly detected in ductal cells.14 Pleomorphic adenomas (PMAs) typically demonstrate positive reactions to calponin, CD9, GFAP, Mcl-2, NM23, p63, S-100, SMA, and Sox10. PLAG1 is a specific diagnostic marker widely employed for identifying PMAs. Occasionally, positive reactions may also be observed for Amylase, DOG1, HMGA-2, KIT, and MYB. In contrast, carbonic anhydrase VI, LPLUNC1, SPLUNC1, and SPLUNC2 are generally negative markers ^6^.

The S100 protein was among the earliest molecules proposed for identifying myoepithelial cells in both healthy and tumorous salivary glands ^15^. Although widely acknowledged as a conventional marker for myoepithelial cells, its positivity has been observed in only a limited number of studies, while appearing negative in other studies examining normal salivary glands. This inconsistency arises from the association of acini and ducts with abundant autonomic nerves, which can potentially be mistaken for S100-positive myoepithelial cells. Alternatively, this variability may be attributed to the heterogeneity within the developing myoepithelial cell population, with some cells potentially representing a pluripotent population ^16^.

Maruyama S, et al., 2009, they also performed a characterization and immortalization of pleomorphic adenoma cells through xenografts, from which they obtained 5 different cell groups: 2 with polygonal cellular characteristics and 3 with spindle-shaped cellular characteristics. S100 immunomarker was applied as a myoepithelial marker, which came out negative in polygonal cells and positive in spindle cells ^17^. This finding is consistent with our results of fusiform morphology and diffuse positivity for the S100 immunomarker, supporting myoepithelial differentiation.

Hou Y, et al., carried out a study culturing in vitro cells of a pleomorphic adenoma through Matrigel, with the aim of differentiating fibroblasts and myoepithelial cells through characterization through groups, the same ones that were he applied the SMA immunomarker, the group of myoepithelial cells were positive to it ^18^. In our study, the cell culture exhibited high marking in the majority of myoepithelial cells.

In contrast, Fujita et al. (1999) conducted three-dimensional culture experiments using pleomorphic adenoma samples. These cultures comprised epithelial cells, mucosal cells, and myoepithelial cells. The groups of epithelial cells tested negative for vimentin, SMA, and S-100, while the groups of myoepithelial cells exhibited the opposite profile, with positivity for SMA, vimentin, and S-100. These findings support our results regarding the characterization of the myoepithelial cells within the subculture, their positive expression of SMA and vimentin confirms their differentiation into myoepithelial cells ^19^. 

Radhika T et al., 2020 carried out a study in which they applied various immunomarkers for the characterization of epithelial and myoepithelial cells. The results indicated positive expression of Vimentin, SMA, Calponin, AE1-AE3, and p63 in myoepithelial cells ^20^. These results reinforce our research findings, as we observed the same immunohistochemical positivity in the myoepithelial cells of paraffin cubes, utilizing the identical immunomarkers as Radhika et al., Additionally, AE1/AE3 has been immunolocalized in liminal epithelial cells of the salivary duct ^21^, where, in our results, luminal cells were positive in our immunohistochemical staining on our paraffin block, and negative in our cell culture. With this result, we can mention that in our culture, there was no growth of luminal cells.

Patrón-Bolaños et al., 2016 conducted a literature review on the stromal variants of pleomorphic adenomas, emphasizing the pivotal role of myoepithelial cells in these histopathological variants. To characterize these cells, the researchers utilized immunomarkers, including Vimentin, and observed positive immunostaining for vimentin ^22^. Vimentin, an intermediate filament, is present in cells of mesenchymal and neuroectodermal origin. It is closely associated with myofilaments and cell adhesion. This implies that myoepithelial cells in pleomorphic adenomas and myoepitheliomas might have the ability to express both ectodermal and mesenchymal cytoskeletal filaments during neoplastic transformation ^23^.

Furthermore, the p63 gene, a member of the p53 family, has been utilized as a specific histochemical marker for myoepithelial cells. It is noted to stain the nuclei of basal or peripheral cells in both normal salivary glands and salivary gland neoplasms ^24^.

## CONCLUSION

The study underscores the significance of cellular morphology in characterizing pleomorphic adenoma. The identification of myoepithelial cells as the primary component, using vimentin and SMA, S100, Calponin, p63 immunomarkers, provides an insight into the nature of these tumors. However, the omission of the specific immunomarkers, such as PLAG1, in this cell culture presents an avenue for future research, promising a more comprehensive understanding. These conclusions emphasize the importance of approaching neoplasm’ complexity with precise methodologies and urge us to continue exploring the various facets of pleomorphic adenomas in pursuit of deeper and more accurate comprehension.
